# Modeling and Theories of Pathophysiology and Physiology of the Basal Ganglia–Thalamic–Cortical System: Critical Analysis

**DOI:** 10.3389/fnhum.2016.00469

**Published:** 2016-09-21

**Authors:** Erwin B. Montgomery Jr.

**Affiliations:** ^1^Greenville Neuromodulation CenterGreenville, PA, USA; ^2^Departments of Neuroscience and Philosophy, Thiel CollegeGreenville, PA, USA

**Keywords:** basal ganglia–thalamic–cortical system, model, theory, globus pallidus interna rate theory, beta-oscillations theory, increased synchronization theory, principles of causational and informational synonymy, logical fallacies

## Abstract

Theories impact the movement disorders clinic, not only affecting the development of new therapies but determining how current therapies are used. Models are theories that are procedural rather than declarative. Theories and models are important because, as argued by Kant, one cannot know the thing-in-itself (das Ding an sich) and only a model is knowable. Further, biological variability forces higher level abstraction relevant for all variants. It is that abstraction that is raison d’être of theories and models. Theories “connect the dots” to move from correlation to causation. The necessity of theory makes theories helpful or counterproductive. Theories and models of the pathophysiology and physiology of the basal ganglia–thalamic–cortical system do not spontaneously arise but have a history and consequently are legacies. Over the last 40 years, numerous theories and models of the basal ganglia have been proposed only to be forgotten or dismissed, rarely critiqued. It is not harsh to say that current popular theories positing increased neuronal activities in the Globus Pallidus Interna (GPi), excessive beta oscillations and increased synchronization not only fail to provide an adequate explication but are inconsistent with many observations. It is likely that their shared intellectual and epistemic inheritance plays a factor in their shared failures. These issues are critically examined. How one is to derive theories and models and have hope these will be better is explored as well.

## The Impact of Theory in the Clinic

Theories, particularly the prevailing theory, have an enormous impact on clinical practice and medical science, the latter often determines clinical practice. Consider the Globus Pallidus Interna (GPi) Rate theory, which posits that overactivity of the GPi suppresses intended movements, resulting in hypokinetic disorders consequent to the inhibitory influence of GPi neurons on neurons of the ventral thalamus pars oralis (Vop). Further, underactivity of GPi neurons results in unintended movements by abnormal disinhibition of thalamic neurons, resulting in hyperkinetic disorders. This theory is demonstrably incorrect, and contrary evidence has been available ever since the initial publications of the theory (reviewed in Montgomery, 2007). Consider the following consequences of the GPi Rate theory.

### Continued Arguments that High-Frequency Deep Brain Stimulation (DBS) Reduces the Output of the GPi, Improving the Hypokinetic Symptoms of Parkinson’s Disease by Reducing Abnormal Inhibition of Vop Neurons

While there is considerable evidence to the contrary, this theory does not explain why high-frequency deep brain stimulation (DBS) also is effective for hyperkinetic disorders. This hypothesis was based on the logical Fallacy of Pseudotransitivity, which presumes synonymy between the neurophysiological mechanisms between DBS in the vicinity of the GPi and subthalamic nucleus (STN) and pallidotomy and subthalamotomy, respectively (Montgomery, 2012). It is important to note that the judicious use of logical fallacies is critical to the advancement of science when its use generates hypotheses for subsequent experimental vindication. However, it is injudicious to use such fallacies to argue in support of any theory or to argue against other alternative theories. In this author’s opinion, this theory has slowed progress in understanding the mechanisms of action of DBS and, importantly, theories of pathophysiology and physiology of the basal ganglia–thalamic–cortical system. It is likely that these arguments also delayed the development of better therapeutic approaches. Indeed, the intuitive appeal of both the GPi Rate theory and the postulated mechanisms of action of DBS became mutually re-enforcing, creating a circularity of explanatory theory, making alternative theories much more difficult to gain traction. At least the consideration of alternatives has the potential of being right.

### Dichotomization of DBS into High Frequencies, Associated with Improvement, and Low-Frequency DBS Associated with Worsening of Symptoms of the Disease Treated

There is considerable evidence to the contrary, yet this notion persists, particularly if insufficient caution and attention are paid to sampling issues with regard to DBS frequencies (Huang et al., 2014). However, the assumption of a dichotomization between high and low frequencies based on the suspect one-dimensional push–pull dynamics of the GPi Rate theory (see earlier discussion) persists (di Biase and Fasano, 2016). The consequence is that the full clinical potential of DBS at a wide range of frequencies has not yet been explored.

### Beyond the Specifics of the GPi Rate Theory, the Underlying Enabling Presumptions are a Dichotomization of the Mechanisms into Two Opposing Contrary Stable States

In the case of the GPi Rate theory, the two states of high neuronal activity vs. low neuronal activity in the GPi dominate theoretical explanations. However, one just as easily could substitute increased beta-oscillatory activity or increased synchronization of the GPi for the overactivity of the GPi neurons in hypokinetic disorders. The one-dimensional push–pull dynamics for which the two states have been the poles that constituted the basis for understanding the effects of various treatments. The one-dimensional push–pull dynamics presupposes the existence to two steady states.

Consider viewing a grayscale that ranges from white to black at the extremes. How many colors (shades of gray) are in the grayscale? Since Aristotle and his notion of the *Contraries* (Montgomery, 2012), the answer continues to be just two—white and black. All the other shades are admixtures of varying degrees of black and white. In the case of the GPi Rate theory, the dimension has overactivity and underactivity at the extremes of the continuum. Disease is occasioned when the actual condition is at an extreme and normality is at some intermediate condition.

The Cholinergic/Dopaminergic Imbalance theory of the movement disorders in the 1970s posited two one-dimensional systems that complemented each other. One dimension was organized on the relative excess or a deficiency of acetylcholine in the basal ganglia. The other dimension had relative excess and relative deficiency of dopamine at its poles. Interestingly, with the ascendency of the GPi Rate theory, any theories to explain the obvious benefit of anticholinergic medications seem to have evaporated, perhaps not unlike the memory holes in the Ministry of Truth in Orwell’s novel *1984* (Orwell, 1949).

The one-dimensional system surviving the Cholinergic/Dopaminergic Imbalance theory was a continuum between relative deficiency and excess dopamine in the striatum, resulting in hypokinetic and hyperkinetic disorders, respectively. The stable states relative to dopamine content thus influenced the notion that dopamine release in the striatum was relatively constant, giving rise to tonic dopamine activity—this despite the demonstration by Schultz et al. (2015) of a very rapid but brief increase in dopamine neuronal activities during a motor task (Schultz, 1986). This theory led directly to dopamine replacement therapies that provided constant application of dopamine (Obeso and Olanow, 2011). However, the failure of tonic dopamine replacement therapy is seen in patients with Parkinson’s disease where increased amounts of presumably tonic dopamine were in the striatum as a result of fetal dopamine transplantation. Many, if not most, patients did not improve. It has been only recently that renewed interest in the dynamic or phasic operations of dopamine neurons has been rekindled (Schultz et al., 2015).

### Presumption of the One-Dimensional Push–Pull Dynamics Influencing Biomedical Research

Research attempting to improve the hypokinetic symptoms of Parkinson’s through genetic manipulation directed at the STN to reverse the state of the STN neurons from one end of the single dimension (excitatory) to the other end of the same dimension (inhibitory) presumes the GPi Rate theory (LeWitt et al., 2011). One might argue “doesn’t the fact that pallidotomy and genetic reversal of the STN neurotransmitter effect demonstrate the validity of the GPi Rate theory?” As will be seen, these evidences do not. Theories that seek vindication by a demonstration of predictions derived from the theories are the Fallacy of Confirming the Consequence. Note that this is not to say that the theory cannot be true, but only that the demonstration of its predictions does not assure that it is true (discussed in greater detail later).

### Presumption of the One-Dimensional Push–Pull Dynamics and Symptoms and Signs in the Clinic

The notion of a one-dimensional dichotomy goes back to Aristotle’s notion of *Contraries* and was fundamental to Galen’s notion of disease. The concept found a home and credibility in the writing of John Hughlings Jackson, called the Father of English Neurology, in his dichotomization of symptoms and signs into positive and negative. Paralysis was a negative sign representing insufficient activity in the motor systems, while seizures and spasms were attributed to excessive activity in the motor systems. Extended to disorders of the basal ganglia, the symptoms and signs were attributed to deficiency and excess of basal ganglia function, respectively. As there is little corroborating evidence sufficient to prove the case, it must be taken as a theory. However, the clinical dichotomization resonates with the GPi Rate theory, as well as with the predecessor and successors of the theory, again demonstrating the intuitive appeal and power of the simple one-dimensional push–pull dynamics.

Parkinson’s disease is considered the archetype of hypokinetic syndromes. The diametric opposite in the one-dimensional dichotomy are the hyperkinetic syndromes, such as Huntington’s disease. It is a testament to the power of theory, particularly those that are intuitive and appealing, that contrary evidence would be trumped. Patients with Parkinson’s disease can have bradykinesia as well as hyperkinesia simultaneously. Similarly, patients with chorea from Huntington’s disease are bradykinetic on volitional tasks.

There likely are many reasons for the power of theories such as the GPi Rate theory and other one-dimensional push–pull theories, such as Beta-Oscillations or Increased Synchronization theories. These range from the polemical, where proponents of popular theories make it difficult for insurgent theories (Kuhn, 1965) by not funding grants, accepting publications, or inviting advocates for alternatives to present, to ways of observing phenomena and adjudicating what is relevant and acceptable evidence (Montgomery, 2012). Abraham Maslov wrote, “I suppose it is tempting, if the only tool you have is a hammer, to treat everything as if it were a nail” (Maslow, 1966). Think of popular theory as a very big hammer.

## Theories Are Necessary but All the More Reason for Diligence

Discussions of theory in biomedical research and clinical science are fraught with difficulty due to misconceptions about the nature of theory. While theory is readily appreciated in physics, chemistry and psychology, often theory is a pejorative term in biomedical research. In the latter, often the presumption is that theory is just so much metaphysical speculation and that data speak for itself. Theory is unnecessary. Disabusing any reader holding this position is beyond the scope of this article. For those readers, this author can only ask forbearance. The position held here is that, at the very least, the facts-of-the-matter, such as observations and evidence as related to the pathophysiology and physiology of the basal ganglia–thalamic–cortical system, are inadequate for a complete explication of the altered behaviors associated with disorders of the basal ganglia–thalamic–cortical system. A certain amount of “connecting the dots” is necessary and thus, the necessity of theory to do so. At the very least, the theoretical connections “between the dots” become the testable hypotheses for subsequent experimentation and thus new knowledge.

Models are a form of theory. Models typically are procedural rather than declarative. They explain by doing but are theories nonetheless. The great advantage is that models can succeed in a procedural sense without the necessity of declarative explications. The latter is discovered *post hoc*. Thus, what is epistemically true of any model is also true of theory.

Science is remarkably effective in the accumulation of facts-of-the-matter. Advances in scientific technology truly are breathtaking, and other fields of human endeavor to discover new knowledge are left wanting. However, truth be told, science is poor at what is most fundamental, that is, the generation of hypotheses that would be subjected to the Scientific Method. This is to be expected as hypotheses necessarily extend beyond the facts-of-the-matter, whether by interpolation or extrapolation, and thus beyond scientific technology. Theories, and their specification in models, require the application of reasoning rather than discovery. Herein lies a problem. While scientists are happy to discuss technology, questioning their reasoning seems beyond the pale. Those that do question reasoning are labeled judgmental and are dismissed. However, any scientific experiment is only as good as the hypothesis it seeks to support or refute. Some scientists just think wrongly and it would be a disservice to look the other way. Any attempts to generate good hypotheses are, first and foremost, exercises in reasoning and not technology.

Whatever theory is, it is not logical deduction and, consequently, does not carry the certainty of deduction. Deduction, either propositional of the form *if **a** implies **b** is true and **a** is true, then **b** is true* or syllogistic deduction of the form *all **a***’s are **b**’s and **c** is an **a**, the **c** is a **b**, provides the highest certainty. The epistemic utility of deduction is that one can be assured true conclusions from a deductive argument with valid propositions and true premises. At the very least, one proposition or premise in any theory cannot be taken as valid or true, respectively, as otherwise the theory would be fact, law, or principle. In an important sense, it is good that theories are not deductions, as deductions do not provide new knowledge, which is the purpose of theory.

Fallacies of deduction can provide for new knowledge as they generate hypotheses that combine to form theories. Indeed, the Scientific Method, when used to assert a positive claim, as opposed to denying the claim, is the Fallacy of Confirming the Consequence. However, as necessary logical fallacies, theories require great caution in their construction and use, as will be demonstrated.

Induction is an alternative that could provide new knowledge but requires presuppositions that risk tautology. For example, if it is observed that every case of increased GPi neuronal activity is consistently associated with Parkinsonism, then one can induce that all cases of Parkinsonism are due to overactivity of the GPi neurons. However, such experience does not preclude the possibility that some case of Parkinsonism are not associated with overactivity of the GPi. The problem is what were the circumstances that allowed observations of overactivity of GPi neurons? If it is limited to high, perhaps excessive, doses of the neurotoxin *n*-methyl-4-phenyl-1,2,3,6-tetrahydropyridine (MPTP), then every examined case using such high doses would have increased GPi neuronal activities. However, as has been known since 1986, one can produce parkinsonism in nonhuman primates, demonstrating neurometabolic changes demonstrated by others as being associated with parkinsonism, without causing the changes in neuronal activities predicted by the GPi Rate theory (Montgomery et al., 1986; subsequently confirmed by Wang et al., 2009). Indeed, the use of dopamine antagonists and electrolytic lesions of the nigral–striatal pathway produced Parkinsonism in nonhuman primates without causing overactivity in the GPi (Percheron et al., 1993).

The same doubts attend the theory that increased beta-oscillations produce Parkinsonism, as 15–20% of patients with Parkinson’s disease do not have increased beta-oscillations. If induction from observations in patients with Parkinson’s disease was used, then the claim is invalid. Note that this is not to say that increased beta-oscillations cannot cause Parkinsonism (hence, a sufficient cause). Rather, the evidence shows that increased beta-oscillations cannot be a necessary cause of Parkinsonism. Similarly, DBS that improves Parkinsonism increases synchronization, which means that it cannot be true that increased synchronization is causal to Parkinsonism. Electroencephalographic-evoked potentials in response to DBS likely would not be detectable if DBS caused desynchronization (Baker et al., 2002; Walker et al., 2012). Similarly, a study of M-wave responses in normal subjects and patients with Parkinson’s disease, with and without DBS, suggests an increased synchronization of lower motor neuronal activity in patients with untreated Parkinson’s disease but even greater synchronization with therapeutic DBS (Aldewereld et al., 2012).

Perhaps the only other alternative is the use of logical fallacies, particularly the Fallacy of Confirming the Consequence and the Fallacy of Pseudotransitivity. The Scientific Method, when used to affirm a claim, is the Fallacy of Confirming the Consequence, which is of the form *if **a** implies **b** is true and **b** is true then **a** is true*. For example, *if overactivity of the GPi neurons* (or *increased beta-oscillations* or *increased synchrony*) *implies Parkinsonism and Parkinsonism is found, then there must be GPi neuronal overactivity* (or increased *beta-oscillations* or *increased synchrony*). It is important to note that had ***b*** in the formal statement been false, then ***a*** would have to be false or the proposition that ***a*** implies **b** would have to be invalid. Thus, if Parkinsonism was not found using methods that produced overactivity, increased beta-oscillations or increased synchrony, then the latter cannot be causal to Parkinsonism. That Parkinsonism was demonstrated is at least a partial victory, assuming that the victory is held with skepticism. Such results are better termed vindication rather than verification.

It would be a disservice to merely point out where some current theories and models regarding the pathophysiology and physiology of the basal ganglia–thalamic–cortical system are inconsistent with or contrary to facts. Rather, it is important to understand the factors that lead to the creation of the theory in the first place, least one repeats the errors. Theories do not emerge fully formed as Athena from the head of Zeus, they have a history. As Santayana (1905) said, “Those who do not remember the past are condemned to repeat it.”

## Metaphors to Advance Understanding of the Physiology and Pathophysiology of the Basal Ganglia–Thalamic–Cortical System

A fundamental question, almost entirely ignored, is where do the hypotheses that constitute theories come from? Very frequently, hypotheses derive from metaphors. For example, it was known that pallidotomy, presumed to silence the output of the GPi, improves Parkinsonism. DBS in the vicinity of the GPi likewise improves Parkinsonism. Thus, the reasonable theory can be constructed that DBS silences the GPi neurons based on the metaphor, pallidotomy is to improved Parkinsonism as DBS in the vicinity of the GPi is to improved Parkinsonism. As pallidotomy silences GPi neurons, DBS in the vicinity of the GPi must silence neurons.

From the metaphor derives a hypothesis that DBS in the vicinity of the GPi should silence neurons. It is now a matter of experimentation to demonstrate the case by recording neuronal activities within the GPi. As this proved to be problematic, the alternative was to use a recording of thalamic neurons, as they would manifest the consequence of increased or decreased GPi neuronal activities. Unfortunately, proposals to do just that found hostility. This is an example where the metaphor relating pallidotomy to DBS in the vicinity of the GPi went from enabling research, as a method of hypothesis generation, to disabling research as the metaphor was used as proof against the alternatives.

## Metaphors and Models

There is a real question of whether any human can ever have a complete and explicit knowledge of the brain given its complexity. All one might know is some approximation of the brain. Thus, at best, all any human can know is some model of the brain and that model must serve as a metaphor for the brain, as the brain itself is unknowable.

Even if it were possible to know everything about an individual’s brain, another’s brain is likely to be different. Even if it were possible to know everything about the brains of each of the two individuals, it would be impossible to know what it is about their brains that leads to an understanding of behaviors in common. One could say that specific arrangements of neurons in person ***A*** result in behavior ***X***. One also could say that a specific arrangement of neurons in person ***B*** also results in behavior ***X***. As those arrangements are different, one would have to either say that there are as many behaviors ***X*** as there are different arrangements of neurons in every individual person or say that there is something in the arrangements of both persons ***A*** and ***B*** that transcends the actual arrangements of neurons in order to produce the same behavior ***X***. The question is what that transcendent entity would look like.

The notion that there is some transcendent entity that accounts for the same functions or behaviors despite entirely different instantiation in the actual neural structure is called Functionalism. The analogy is that the same word processing operations can be implemented in a variety of hardware. The intriguing question is whether that transcendent entity is a better explanation of brain functions than the actual neural physiological architecture in any actual instantiation. Then would not modeling of the transcendent entity have a higher probability of providing insight than explicit examination of each instantiation in each individual?

Whether an explicit understanding based on the exact instantiation of neuronal physiologic architecture is not possible or whether the transcendent functional entity is a better target for understanding, the net result is that only modeling is possible. Modeling is the only option to gain any understanding.

Models are used as metaphors—*the operations are to the brain as are the operations are to the model*. Metaphors contain a target and a source domain. The target is the statement that needs explication, while the source domain is the statement that suggests an explication. For example, consider the metaphor *DBS is to improvement of Parkinsonism as pallidotomy is to improvement*. The source domain *pallidotomy is to improvement* provides a suggestion as to the nature of the target domain, *DBS is to improvement*. Whatever mechanisms by which pallidotomy produces improvement are used to suggest the mechanisms by which DBS provides improvement. While this is not verification, at the least it offers a hypothesis for subsequent experimentation.

Every metaphor contains epistemic risk, that is, the metaphor may lead to a wrong hypothesis. The mechanisms underlying pallidotomy need not be the same as underlying DBS and, indeed, they are not. Thus, any modeling is only as good as the enabling metaphor. The question is how to evaluate the potential of any metaphor, and thus its epistemic risk.

Epistemic risk involves epistemic distance and epistemic degrees of freedom. Epistemic distance is the degree of dissimilarity between the target domain and the source domain. For example, consider the metaphor *kainic acid injections in the GPi are to improvement as pallidotomy is to improvement*. Although, to the knowledge of this author, kainic acid lesions of the GPi have not been studied for its ability to improve movement disorders improved by pallidotomy, the hypothesis that kainic acid injections into the GPi may improve movement disorders is not unreasonable and suggests that experimental vindication may be worthwhile. The epistemic distance between pallidotomy, which destroys all the neuronal elements in the target, including neuronal cell bodies, as well as axons in passage, is not that different from kainic acid lesions that destroy the neuronal cell bodies, sparing (relatively) axons in passage.

Epistemic degrees of freedom relate to how many modifications would be required to increase the similarity between the target and the source domain. Consider the metaphor *DBS is to improvement as pallidotomy is to improvement*. The metaphor seeks to “equate” DBS to pallidotomy. Pallidotomy results in the wholesale destruction of neuronal elements at the site. DBS involves applying electrical pulses to the site. The question is how many twists and turns of logic would be required to make wholesale destruction equivalent to applying electrical pulses? This is very difficult because virtually every other experience of applying electrical stimulation has been to excite rather than suppress neuronal activity (Montgomery and Baker, 2000). Even studies that demonstrate a reduction of action potentials back propagated into the soma likely are due to the activation of presynaptic terminals that release neurotransmitters, which result in hyperpolarization of the postsynaptic membrane. Thus it is not easy to translate electrical stimulation to inhibition of neuronal activity as the link to pallidotomy would suggest. The epistemic degrees of freedom would have to be very high, thereby resulting in a high degree of epistemic risk when using the metaphor. That high epistemic risk has been borne out by observations that DBS does not result directly in suppressing neuronal activity at the neuronal membrane. DBS can generate action potentials at the initiating segment even if activation of presynaptic terminals result in hyperpolarization of the soma. Certainly, the direct physiological effects of the changes in the electrical fields involve a variety of voltage gated ionic conductance changes, such as sodium and calcium channels, as well as affecting NMDA receptors (extensively discussed in Montgomery, 2016).

## Epistemic Risk and Aristotle’s Principle of Causational Synonym

Epistemic risk, based on epistemic distance and degrees of freedom, is a refinement of Aristotle’s Principle of Causational Synonymy. This principle holds that whatever mechanisms are contained within a cause must be the same as the mechanisms contained in the effect. For example, consider how the movement of a hand in a pool of water can cause the water to move. The hand is solid and the water a liquid. It may not seem that there is any causational synonymy between the hand and the water. However, the electrons in the orbits around the atoms of the surface of the hand repel the electrons in the orbits around the molecules of water. Thus, there is clear synonymy in terms of the repulsive forces between electrons.

If one accepts that the Principle of Causational Synonymy applies to causal models of pathophysiology of movement disorders and the physiology of the basal ganglia–thalamic–cortical system, then current popular models fail. The GPi Rate and the beta-oscillations theories hold that there is a gate-like mechanism and that the pathophysiology of Parkinson’s disease is due to the gate being closed excessively. This notion is clear and direct in the GPi Rate theory, where the overactivity of neurons of the GPi shut down activity in Vop and consequently in the motor cortex. The excessive beta-oscillatory activity is thought to prevent activations in the remainder of the basal ganglia–thalamic–cortical system to produce movement. Thus, the cause is some steady-state manifest by excess GPi neuronal activities or beta-oscillations.

The effect is not at all a steady state. Parkinsonism is not the relative absence of movements, automatic (habitual) or otherwise. Rather, the movements themselves are abnormal. The normal recruitment order of motor units, with small units activated first, followed by progressively larger motor units, is disrupted in Parkinson’s disease. The normal triphasic pattern of electromyographic activities associate with rapid ballistic movements is abnormal. The normal reciprocal activities between agonist and antagonist are disrupted. Higher level synergies of muscular activities over multiple joints with complex movements are abnormal (for a review, see Montgomery, 2013). Thus, very little synonymy exists between the mechanisms of pathophysiology of the basal ganglia attributable to these prevailing theories and the mechanisms by which motor unit orchestration is disrupted. The dynamics of the theories are one dimensional and push–pull. The actual dynamics represented by motor unit activities are far more complex.

The great problem for those attempting to explicitly model activities within the basal ganglia lies in the dynamics that the models are intended to capture. If these models are based on currently popular theories, then the models are going to be one-dimensional push–pull dynamics. A great number of such models have been offered typically under the notion of Actor–Critic models.

## Principle of Informational Synonymy

The Principle of Causational Synonymy can be extended to the Principle of Information Synonymy. One can define information as nonrandom changes in states, either in parallel or in sequence. The patterns of motor unit recruitment in normal movement are not random and therefore contain information. Further, information contained in the pattern of motor unit recruitment and de-recruitment must be contained in the information provided to the lower motor neurons, although the information may be altered based on the properties of the lower motor neuron.

The classical notion is that the motor cortex—the predominant source of information to the lower motor neurons, particularly those that project to the distal musculature—specifies the time course and the magnitudes of the forces to be generated. The biophysical properties of the lower motor neurons then convert that information into the orchestration of motor unit recruitment according to the Henneman Size Principle. However, studies have demonstrated that Parkinson’s disease disrupts the orchestration of motor unit recruitment, violating the Henneman Size Principle, which is normalized with therapeutic DBS (Huang et al., 2012). Thus, any model of the function of the basal ganglia–thalami–cortical system must account for the orchestration of motor unit recruitment.

The GPi Rate, the beta-oscillator, and the increased synchronization theories do not account for these dynamics and hence cannot be considered adequate. The information encoded in the basal ganglia–thalamic–cortical system, perhaps with other descending inputs to the lower motor neurons, must be at least equal to the information contained in the orchestration of motor unit recruitment and de-recruitment. It cannot be the case that the lower motor neurons themselves create additional information, as this would be a violation of the Second Law of Thermodynamics as applied to information. The Second Law of Thermodynamics holds that in any closed system, entropy (considered the converse of information) cannot decrease. This means that in any closed system, net information cannot increase. Information in the activities of the lower motor neuron that drives motor unit activity cannot be generated from the lower motor neuron independently of its inputs, such as from the basal ganglia–thalamic–cortical system. What can occur is either a loss of information, perhaps manifesting as disease, or a conversion of information from one form to another that preserves the overall information content. One-dimensional push–pull dynamics, such as underlie the GPi Rate, beta-oscillator, and hypersynchronization theories, are just insufficient.

## Models and Metaphors Needed that Approximate the Dynamics and Information of the Systems Modeled

Any theory, model, or metaphor whose fundamental dynamics are one-dimensional push–pull will be inadequate. Certainly, such simple models are more tractable and one might argue that is a place to start. The program is to start with these simple one-dimensional systems and to increase the complexity progressively. Thus, the models can extrapolate from the simpler precursors in an incremental manner. Unfortunately, this will not work and holding to this incrementalist agenda is only likely to delay significant breakthroughs. Experience with other physical systems demonstrates that at some point in increasingly complex models, the quantitative evolution becomes a qualitative revolution in which the prior inferences are unlikely to hold.

Consider Newton’s laws of motion and gravitation. These apply well to single bodies, such as an object in motion not subject to external forces. They apply equally well to two body problems, such as the moon orbiting the earth. However, these laws fail in their ability to predict the three body problem precisely, such as the orbit of the moon about the earth and both orbiting around the sun. Rather, this three body problem demonstrates Complexity, as first suggested by Henri Poincare. The fundamental property of Complex Systems is their unpredictability. At some point, increasing complexity incrementally likely will result in a qualitative change where inferences from preceding systems that are just an iota less complex fail to predict and thus explain the behavior of the complex system.

The facts just described mean that a reductionist approach, in empirical as well as in theoretical science—which includes modeling—is profoundly limited. Rather, in Complex Systems, such as would describe the pathophysiology and physiology of the basal ganglia–thalamic–cortical system, one needs to begin with the complex system. Modeling must look to examples of Complex Systems for the metaphors that will inform future modeling endeavors, which will be critical to any subsequent successful empiric understanding. There are beginnings, such as models based on networks of loosely coupled polysynaptic re-entrant nonlinear discrete oscillators (Montgomery, 2004, 2007). While it is far too soon to know whether this work will prove to explicate the complex dynamics underlying the pathophysiology and physiology of the basal ganglia–thalamic–cortical system, at the very least it represents a radical departure from the one-dimensional push–pull dynamics of popular conceptions.

Unfortunately, many recent models continue to view the basal ganglia–thalamic–cortical system as a sequential processing system rather than in parallel (Schroll and Hamker, 2013), which likely is an error (Montgomery and Buchholz, 1991). It is interesting that in their review, Schroll and Hamker (2013) describe both open loop, perhaps analogous to sequential processing, and closed loop, which would not be analogous to sequential processing given the dynamics of information transfer within the basal ganglia–thalamic–cortical system. However, they and others fail to appreciate or explore the implications for information processing within the basal ganglia–thalamic–cortical system in the context of behavior.

**Figure 1:**
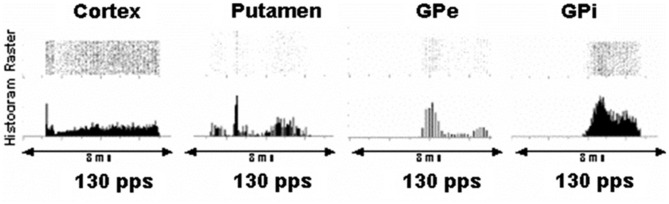
**Peri-event rasters and histograms of a single representative neuron in the cortex, putamen, globus pallidus externa (GPe) and globus pallidus interna (GPi) in the interpulse stimulation period during deep brain stimulation (DBS) in the vicinity of the subthalamic nucleus (STN).** Each row in the raster is the response following a single DBS pulse. Each dot represents discharge from the neuron. Columns created and summed across the peri-event raster result in the histogram, which demonstrates average responses over time. The microelectrode signal was sampled at 25 kHz. The discrimination of the microelectrode recordings into spike waveforms indicative of individual neurons was confirmed by demonstrating a refractory period in the autocorrelogram and absence of a refractory in the cross-correlogram among pairs of neurons simultaneously recorded. As can be seen in the cortex, there is an abrupt short latency response at approximately 1 ms following a pulse from DBS in the vicinity of the STN consistent with antidromic activation of cortical axons projecting to the STN. At 2.5 ms there is a moderately consistent increase in activity in a neuron in the putamen. The consistency suggests a monosynaptic input via axon collaterals from cortical neurons activated antidromically. Broad peaks beginning at 3.5 and 4 ms are noted in the GPe and interna, respectively, suggesting orthodromic activation of axons from the STN projecting to these nuclei (modified from Montgomery and Gale, 2008).

As shown in Figure 1, effects of a DBS pulse in the vicinity of the STN causes responses with very short and highly consistent latencies in the cortex consistent with antidromic activation of cortical projections to the STN. There is a response in putamen neurons at approximately 2.5–3 ms. This likely represents orthodromic activation of putamen neurons, and one source would be collateral branches of antidromically activated cortico–subthamic nucleus axons that project to the putamen. Similarly, there are responses in GPi and externa at 4 and 3 ms, respectively, which are perhaps monosynaptic activations from stimulated STN neurons.

The key point is that information transfer between nodes within the basal ganglia–thalamic–cortical system is very fast, on the order of 3–4 ms. If a bit of information is generated in the motor cortex, it would activate putamen neurons 3 ms later and STN neurons at 3 ms. GPi neurons would be activated at 7 ms, which then would produce posthyperpolarization rebound in neurons of the Vop at 10.5 ms and then back to the cortex at 14 ms. Next consider a movement that is executed over 1 s. This means that the motor cortex can drive the remainder of the nodes 71 times during the course of the movement. It also means that any structure within the basal ganglia–thalamic–cortical system can drive the motor cortex 71 times during the behavior. Further, the motor cortex, for example, receiving information through what is called the indirect route, could send information to what is called the direct route or what is called the hyperdirect route 71 times during the course of a 1-s behavior. Thus, the time course of information percolating though the basal ganglia–thalamic–cortical system relative to the time course of the behavior, called the duty cycle, is very small. The analogous situation is the fact that each pixel on a computer screen is “painted” sequentially but is so fast relative to human perception that events appear simultaneously on the screen. Clearly, given these dynamics, does it make sense to talk or model the basal ganglia–thalamic–cortical system as separate and discrete pathways operating as open loops in a sequential manner?

## Modeling to Normal from Abnormal and Teleological Thinking

The problematic nature of inferring normal function from alternations in the normal subject was well known to the ancient Greeks and a substantial reason for the reticence toward vivisection. Research involving lesions in animals is a study of abnormal animals. One cannot assume that inferences from lesioned animals or disordered humans will translate easily into an understanding of normal function. The British neurologist Francis M. R. Walsh in the early 1900s likened the situation to the circumstance of gear teeth in the differential gear of an automobile breaking and causing a clunking sound. It would be an error in reasoning to then think that the purpose of the differential gear is to prevent clunking sounds.

It would be a similar misreasoning to infer that because disorders such as a stroke involving the STN result in involuntary movements that the function of the STN, specifically, and the basal ganglia–thalamic–cortical system is to prevent involuntary movements. There are alternative explanations (see Montgomery and Baker, 2000). However, just such a presumption is made in many of the so-called Actor–Critic models of the basal ganglia–thalamic–cortical system. These Actor–Critic models also presumed the highly improbable theory proposed by Mink and Thach (1993), whose only support appears to be lifting the center-surround antagonist physiology of the visual system. A critical review is beyond the scope of this article, but it is suffice to point out that cross-correlation studies of neurons recorded within various structures of the basal ganglia–thalamic–cortical system fail to demonstrate the type of negative correlation that would be reasonably expected according to the model proposed by Mink and Thach (1993).

Teleological thinking and its attendant problems are epidemic in models of the basal ganglia–thalamic–cortical system, as can be seen in the review of computational models by Schroll and Hamker (2013). The method is to begin with the putative purposes of the basal ganglia–thalamic–cortical system and then to demonstrate that the model behaves in a manner consistent with the putative purposes. With all due respect to Aristotle, the champion of such teleological thinking, and his subsequent conceptual heirs, this reasoning is to put “the cart before the horse.” The problems that ensue have been alluded to previously.

## The Myth that Observations Or Data Speak for Itself and the Logic of Modeling

Unlike physics originally and chemistry historically later, biology has not fully embraced formal modeling, particularly in its quantitative or mathematical sense. There may be many factors, such as it is difficult to explicitly describe complex biological phenomena in such a way that the mathematics give some intuitive sense of the underlying reality, particularly in a mechanistic sense. Also, there may be a presumption that mathematical explication is unnecessary, as the data “speak for itself.” Upon careful epistemological analysis, such a presumption is a myth, but further discussion of the mythological nature is beyond the scope of this article. However, if data did “speak for itself, ” there would be no need of theory. As discussed previously, there clearly is a need for theory.

In the case of popular current theories of the pathophysiology and physiology of the basal ganglia–thalamic–cortical system, it may be that the simple one-dimensional push–pull dynamics appear not in need of mathematical explication. This does not mean that mathematical modeling has not been done for the basal ganglia–thalamic–cortical system. Rather, the mathematical modeling generally is a demonstration or proof of concept. These models demonstrate that when organized in the right way with the appropriate initial conditions, the behavior of the model will be analogous to what the pre-existing theories predict. This reasoning suffers from a conjunction of two fallacies. First is the Fallacy of Confirming the Consequence, which in this case is constructed as *if **model A of parkinsonism** implies **increased neuronal activity in the GPi and increased neuronal activity in the GPi** is found, then **model A is true of parkinsonism***. The same outcome attends having ***b = increased beta oscillation*** or ***b = increased synchronization***. The discussions provided previously prove these arguments false. Note that the goal of modeling is not necessarily to demonstrate increased GPi neuronal activity, beta-oscillations, or increased synchrony. This would be, at the very least, an empty exercise and, at the very worst, sophomoric and misleading. The argument could be recast as *if and only if model **A*** implies **increased neuronal activity in GPi** and** increased neuronal activity in GPi** is true then model **A** is true. Note the claim relating to *model **A*** to Parkinsonism has been dropped. This model, if demonstrated empirically (*in vitro*, *in vivo*, or *in silico*), would be interesting and informative. The next important question is what is it about *model **A*** that it only can result in increased activity in the GPi?

A major problem with the very large majority of modeling of the basal ganglia–thalamic–cortical system has been models presuming a kind of *if and only if* mentality in the context of one-dimensional push–pull and sequential dynamics. Increased neuronal activity in the GPi, necessarily and only, results in decreased neuronal activity in Vop. Yet, this is not true. To be sure, there is an initial reduction in Vop neuronal activity with DBS in the vicinity of the GPi activating GPi efferent axons, but in the majority of cases, there is posthyperpolarization rebound excitation. The posthyperpolarization rebound results in a net increase in neuronal activity for many neurons. Added to this is a further increase in neuronal activity likely from positive feedback from activated cortical neurons projecting back to Vop neurons (Montgomery, 2006).

Ignoring or ignorance of these complex additional dynamics risks the Fallacy of Limited Alternatives. This fallacy is of the form *if **a** inclusive-or **b** inclusive-or **c** is true and **b** and **c** are found false then **a** is true*. (In Probability theory this fallacy is known as the Gambler’s Fallacy.) Note the use of the *inclusive-or*, which allows any or all of the entities be true so long as one is true. Thus, the truth or falsehood of ***a*** is independent of ***b*** and independent of ***c*** and ***b*** is independent of ***c***. Also, note that with ***b*** and ***c*** removed for being false, the result is the Fallacy of Confirming the Consequence. Failure to consider that the actions of GPi neurons on Vop neurons are more akin to delayed excitation rather than inhibition would result in the Fallacy of Limited Alternatives.

The Fallacy of Limited Alternatives is a tremendous challenge to mathematical and computational modeling. The mathematical and computational tools are extremely powerful. For example, genetic and neural network computing do not even require any *a priori* knowledge of how the computational solution should be arrived at. Even if one were to constrain the degrees of freedom of a proposed computational modeling by prescribing that the neural elements of the computational process obey the constraints found in biological neurons, the process is still incredibly powerful and can produce a great variety of computational processes that arrive at the same solution (Marder and Taylor, 2011).

The great, perhaps greatest, value of modeling is the demonstration that the model has to be the one to predict the behavior of interest, not that it could predict the behavior. The reason is that demonstrating that a model has to be the one that predicts the behavior greatly increases the likelihood that the model explicates the behavior. Certainly, in practice, it may not be possible to say that one and only one model can predict the behavior. However, the effort should be to minimize the number of candidate models and then search for commonalities and differences in the dynamics of the model to find those mechanisms of the model that likely are explanatory, following from Mill (1843) Joint Method of Agreement and Difference for Induction.

It is worth considering why modelers have been so susceptible to the Fallacy of Limited Alternatives. At least one answer is that the susceptibility is in the language. The vernacular has terms such as excitation and inhibition, which are incomplete and consequently misleading. Rather, depolarization or hyperpolarization should be used instead. Hyperpolarization at least connotes the possibility of posthyperpolarization rebound excitation, and depolarization connotes the possibility of a depolarization blockade. Further, the one-dimensional push–pull mental predisposition is re-enforced by conflating neurotransmitters with actual electrophysiological dynamics (Valenstein, 2005). Neurotransmitters are the messenger, not the message. To hold that the neurotransmitter is the determinant of neural functions, and thus neural functions can be inferred from neurotransmitters, is like saying the operations of a computer can be inferred directly from the properties of an electron. Note that this is not to say that electrons are not the fundamental element that underlies electronic computers, but it is the same electrons that underlie televisions and smartphones. Thus, the properties of an electron are necessary but not sufficient to understand the operations of a computer. Most neurological and psychiatric disorders are disorders of information. Neurotransmitters (and gap or electric junctions) are necessary but insufficient for information or misinformation. Holding that neural behaviors can be inferred from the actions of specific neurotransmitters would be an example of the Mereological Fallacy (see Montgomery, 2012).

Similarly, talk of the pathophysiology and physiology of the basal ganglia–thalamic–cortical system, and thus by extension, neural models, is couched in terms of neurons. However, use of the term *neuron* carries the connotation that the neuron is the fundamental unit, anatomically, and by extension physiologically. This similarly represents a Mereological Fallacy.

## Reductionism and the Need for New Metaphors

Reductionism often is a choice when one wants an understanding of complex behaviors that goes beyond purely descriptive (sometimes referred to as the “stamp collecting” approach to science). The choice to go beyond the merely descriptive necessitates that the set of behaviors to be understood (the explanandum) must have a set of explanations (explanans) with fewer elements. If the required elements of the set of explanans equaled that of the set of explanandums, the consequence would be purely descriptive. Thus, reductionism is the necessary consequence. Further, in science the relationship between the explanans and the explanandum typically is causal in nature.

Mathematical and computational modeling is no different. Even when employing methods such as genetic and neural network computing—methods that are not necessarily reductive—once the computational solution is determined, it is “dissected” to understand how its “components, ” sometimes referred to as motifs in network or systems theory (Alon, 2007), represent an economical set of causal mechanisms.

Reductionism is relatively easy—all one needs, metaphorically speaking, is a bigger hammer, sharper knife, or more powerful computer. The value of reductionism lies not in the reduction, but in the reconstruction from the economical set of explanans; a notion often forgotten or never appreciated by scientists. The goal is to understand the behavior; an understanding the reduced preparation, be it a tissue slice or a mathematical or computational model, is the means, not the ends, at least in the context of any understanding of the basal ganglia–thalamic–cortical system with the purpose of effecting a benefit in the clinic. Even aside from utilitarian considerations, studying a “reduced” preparation on its own is not synonymous with the notion of reductionism.

A successful reductionism is when the explanandum can be reconstructed from the explanans. One could reduce, methodologically, the basal ganglia–thalamic–cortical system to a system of neurotransmitter fluxes, but it is highly unlikely that one could explicitly reconstruct the normal and abnormal orchestrations of motor unit behaviors in health and disease. The failure of any reconstruction, defined by deficiency or error, indicates that information was lost. If the method by which information was lost is irreversible, then by the Second Law of Thermodynamics as applied to information, the reduction will never allow a reconstruction.

In the case of the basal ganglia–thalamic–cortical system, one such reduction is to view the system as a sequential hierarchical system. This is implicit in all descriptions of the system where the putamen is viewed as the input and the GPi and substantia nigra pars reticulata are viewed as the output. However, an alternative conception is that the basal ganglia–thalamic–cortical system is a set of loosely coupled polysynaptic re-entrant nonlinear discrete oscillators for which there is considerable supporting evidence (Montgomery, 2016). Assuming that the theory is true, then reducing the basal ganglia–thalamic–cortical system to a sequential hierarchical system will eliminate the important re-entry oscillator dynamics. Consequently, any theory based on a sequential hierarchical organization will be unable to ever reconstruct the behavior due to the basal ganglia–thalamic–cortical system.

The critical question then becomes what is the nature of a reconstruction? It may be a matter in practice or in principle that a full reconstruction is impossible; more on this subsequently. However, one might be able to appeal to the possibility or approximation of such a reconstruction. For example, it may just be impossible to reconstruct, in a fully explicit way, the behavior of any individual given the biological variability and complexity of the nervous system. However, one can make “approximations” by appealing to the average of a set of explanans and set of explanandums, with the presumption or assumption that the average represents the Central Tendency; the latter itself is a problematic notion—witness the distinction among mean, median, and mode as they vive for the claim of the Central Tendency.

Perhaps the Reductionism counterpart to the average is the asymptote. In this case, reconstruction using the economical set of explanans converges onto the explanandum. In other words, the limit of the reconstruction as the effort and sophistication approximates perfection becomes the reconstruction. This is analogous to limit theory used in differential calculus where some value ***y*** becomes ***dx/dt*** as ***dt*** approaches zero, for example, where ***y*** is the instantaneous velocity, ***dx*** is the change in distance, and ***dt*** is the change in time.

Another example is Galileo’s demonstration of inertia using inclined planes. A ball rolling down an inclined plane will roll up another incline plane to the same height from which it descended. As the angle of the second incline is reduced, the ball has to roll further along the incline to reach the same height. As the second incline is continually reduced, the ball rolls further. The presumption is that, independent of friction, at a zero angle on the second incline, the ball would roll forever; hence, Galileo’s law of inertia. Yet, clearly it would be impossible to demonstrate this fact empirically. Consequently, Galileo’s argument forever would be a metaphor, but nonetheless a very convincing one. This argument is a Process Metaphor. In this case the target domain, that being Galileo’s law of inertia, gets its credibility from the source domain, the experiment of successively reducing the angle of the second inclined plane.

The Process Metaphor is endemic to modeling, particularly mathematical and computational. Typically, one begins with the neuron as a model, its dynamics described by Hodgkin and Huxley constructions of voltage and ligand-gated ionic conductance channels or other abstractions that are more computationally tractable, such as the FitzHugh–Nagumo model or simpler integrate-fire neuron models. One then progressively increases the complexity of the models with the expectation that the model outputs will converge on a dynamic or mechanism that can be thought representative of the underlying biology. As it is unlikely that even an infinitely complex model, defined here as the practically infinitely complex, then the credibility of the model is dependent on the manner in which the model was made more complex, that is the Process Metaphor.

## Failure of Reductionism and Chaos and Complexity

There are notable examples where a Reductionist account has failed to reconstruct the behavior of interest. For example, using Newton’s laws of motion and gravitation, one can explicitly determine the motion of one planet about another, such as the moon about the earth, but not about the moon orbiting the earth as both orbit the sun, as described earlier. This is called the Three Body Problem, which Henri Poincare, and others, demonstrated that no analytical solution was possible, although the system could be approximated later and, in some analyses, an asymptotic solution is possible. Poincare’s analysis was one of the first indications of the unique situation of Chaos.

It is important to realize that Chaos and Complexity, which came along later, are not random systems. They are determinant in that they are based explicably on specific laws and principles. For example, the formation of a snowflake is an example of a Complex System. One of the hallmarks of Complex Systems is their unpredictability. Indeed, the actual geometry, other than having six points, of any particularly large snowflake (containing very large number of water molecules) is different from any other. Thus, it would be hard to predict any particular large snowflake, although mathematical and computational modeling of the growth, as distinct from origin, is advancing (Barrett et al., 2012).

Many chaotic and complex systems do demonstrate “structure, ” hence information. Note that the structures of snowflakes are not random. How “structure” arises and how it can be recognized in chaotic and complex systems is the challenge (see Strogatz, 2014). The structure in these systems may be evident in various “attractors” and bifurcations. Other examples of the dynamics of chaotic and complex systems include a dependence on initial conditions and bifurcations to and between metastable states.

Whether or not the basal ganglia–thalamic–cortical system is a chaotic and complex system remains to be determined. According to at least one theory, called the Systems Oscillators theory, the basal ganglia–thalamic–cortical system has all the “ingredients” for a chaotic and complex system, including the highly nonlinear dynamics of discrete oscillators and the vastness of the potential interactions. There is some preliminary supportive evidence (Montgomery, 2016). However, what is clear is that any reductive method or reduced theory is not going to be able to demonstrate chaos and complexity, and thus not be able to leverage the dynamics of chaotic and complex systems for an adequate theory of the pathophysiology and physiology of the basal ganglia–thalamic–cortical system. At the very least, the Chaotic and Complex Systems theory should be considered a metaphor for further research, particularly modeling, of the basal ganglia–thalamic–cortical system.

Developments in physics, mathematics and physical chemistry are advancing dramatically, as evidenced by the Chaos and Complexity theory. There are a number of other physical–mathematical concepts that, if appreciated by neuroscientists, could advance our understanding of the basal ganglia–thalamic–cortical system greatly. It always is hard to predict which nascent concept will have future impact—witness the initial tepid reception to the transistor and personal computer. Potential areas for fruitful scholarly efforts lie in discrete oscillators (not the kind that use digital signal processing to generate oscillators that approximate continuous harmonic oscillators), as these are more realistic in biological neural oscillators (Montgomery, 2016). Another area that could prove illuminating is nonequilibrium steady states, particularly as how they may underlie metastable states associated with chaotic and complex systems.

## An Alternative Model for the Purpose of Demonstrating Contrasts with Current Models

The Systems Oscillators theory (Montgomery, 2016) begins with the same loop-like architecture that underlies most anatomical concepts of the basal ganglia–thalamic–cortical system but closes them into reentrant oscillators. In doing so, it becomes clear that the basal ganglia–thalamic–cortical system can be seen as a network of loosely coupled reentrant oscillators. Each oscillator contains nodes defined as sets of neurons in the various anatomical structures that make up the basal ganglia–thalamic–cortical system. Neurons within a node are defined by their shared inputs and projections to the same set of neurons in the subsequent node. Different oscillators can share the same nodes thus resulting in a network of coupled oscillators.

The next conceptual difference is that the Systems Oscillators theory addresses the dynamics of neuronal activities at relevant time scales. For example, based on a 3.5 ms time for a “bit of information”, such as an action potential, to pass from one node to the next, the time to traverse a four node loop, such as motor cortex–putamen–GPi–Vop, is in the order of 14 ms. This corresponds to a reentrant oscillator frequency of 71 Hz. This means that the motor cortex can influence the GPi 71 times during the course of a 1 s behavior. Similarly, the GPi can influence the motor cortex 71 times. Thus, does it make any sense to call the putamen an input stage or the GPi as an output stage? Further, information processing does not occur in a manner restricted to a given anatomical structure. Thus, does it make any sense to ascribe a “behavioral function” to any specific anatomical structure, such as the GPi? Does it make any sense to construct a model with structures identified as input or output stages or with anatomical structure assigned a specific behavioral function? The dynamics also demonstrate that a bit of information received by the motor cortex that came through the globus pallidus externa (GPe) could affect the putamen or the STN and then the GPi many times during the course of a behavior. Does it make sense to talk about the physiology unique to the direct, indirect or hyperdirect pathways?

Finally, while most of the neurotransmitters involved in the anatomical connections within the basal ganglia–thalamic–cortical system utilize GABA and produce hyperpolarizations, research has shown that such hyperpolarizations are followed by rebound excitation. In some cases, the rebound excitation results in a net increase in action potential generation, not a decrease. Therefore, does it make sense to talk about inhibitory actions within the basal ganglia–thalamic–cortical system? It makes little sense in modeling the basal ganglia–thalamic–cortical system in terms of excitation and inhibition. The dynamics are far more complex and because of the oscillators, dynamics more likely reflect positive and negative stochastic resonance, stochastic coherence, phase changes, phase entrainment and “noisy” synchronization. Perhaps modeling of the basal ganglia–thalamic–cortical system might benefit from incorporating such dynamics.

Evidence supporting these conceptualization are addressed and more extensive discussion is provided elsewhere (Montgomery, 2016).

## Summary and Future Directions

Modeling of the basal ganglia–thalamic–cortical system is fundamental and critical for many epistemic reasons. Modeling, considered as a procedural form of theory, helps to understand when direct knowledge is not possible because of incomplete direct knowledge. Ideally, it is the models that generate strong hypotheses that, once vindicated by experimentation, expands knowledge. Indeed, it can be argued reasonably that models are the only route to any understanding.

Modeling for the intent of generating hypotheses necessarily requires the judicious use of logical fallacies. Injudicious use not only leads to flawed models but also inhibits development of new models. Further, models and other forms of theory to not arise spontaneously but are legacies of prior models and theories and risk inheriting many of the presuppositions and misconceptions. Among the misconceptions are dynamics that are one-dimensional push-pull and conflating the hyper- and depolarizing effects of neurotransmitters with inhibition and excitation respectively. Also, the Mereological fallacy of attributing to the part, the function of the whole is rampant as is reductionism.

The primary but unappreciated value of reductionism is not the ability to reduce phenomena to simpler forms, but rather in the ability to reconstruct the phenomena from the reduced forms. However, there is a fundamental limit to the ability to reconstruct based on Chaos and Complexity theory. At some point incremental increases in complexity go from a quantitative to a qualitative change at which point the inferences from the just slightly simpler reconstruction is no longer applicable. Thus, the ultimate goal is to have a model whose complexity is at a level of complexity on par with the phenomena the model is intended to explicate. It is not clear that a reductionist approach will be successful.

If one wants to change the future, in this case to a future of greater knowledge and understanding, then one must know the present. To know the present, one must know the past. The future of modeling will not likely succeed if it inherits misconceptions. Modelers of the future will need to rid themselves of the misconceptions and look to new metaphors by which to fashion future hypotheses and theories. Fortunately, rapid advances in physics and mathematics can help, such as Chaos, Complexity, Percolation theory, nonequilibrium steady states and networks of discrete nonlinear oscillators.

## Author Contributions

The author was responsible for all aspects of the article.

## Funding

This effort was fully funded by the Greenville Neuromodulation Center, Greenville, PA, USA.

## Conflict of Interest Statement

The author declares that the research was conducted in the absence of any commercial or financial relationships that could be construed as a potential conflict of interest. The reviewer SC and handling Editor declared their shared affiliation, and the handling Editor states that the process nevertheless met the standards of a fair and objective review.
